# Post-Traumatic Radial Artery Pseudoaneurysm at the Wrist Level: A Case Report

**DOI:** 10.7759/cureus.52875

**Published:** 2024-01-24

**Authors:** Shreosi Sarkar, Ansarul Haq, Veena K Singh, Varun H Kashyap

**Affiliations:** 1 Department of Burns and Plastic Surgery, All India Institute of Medical Sciences, Patna, IND

**Keywords:** vena comitantes graft, surgical repair, wrist level, pseudoaneurysm, radial artery, post-traumatic

## Abstract

A pseudoaneurysm is a collection of blood outside the arterial lumen but remains in continuity with the lumen and lined by fibrous tissue. Radial artery pseudoaneurysm is a rare entity mostly occurring due to iatrogenic reasons. Traumatic causes are rare. In this case report, we report a post-traumatic left radial artery pseudoaneurysm at the wrist level in a 20-year-old male patient. The patient was treated with end-to-end repair of excised pseudoaneurysm with a vein graft taken from the radial artery vena comitantes through the same incision.

## Introduction

A collection of blood outside the vessel wall communicating with the arterial lumen and lined by a single layer of fibrous tissue is called a false aneurysm/pseudoaneurysm. It is formed following an injury to all the layers of an artery [1]. It can be iatrogenic, post-infectious, or post-traumatic. A pseudoaneurysm of the radial artery is an infrequent location of peripheral artery aneurysm. It mostly occurs after transradial cardiac catheterization or other therapeutic procedures, such as arterial blood gas analysis (ABGA)/intra-arterial blood pressure monitoring. A post-traumatic radial artery pseudoaneurysm is even rarer. There are many non-surgical and surgical treatments but no standard guidelines. Here, we present a case of a 20-year-old male with a one-month-old post-traumatic left radial artery pseudoaneurysm who was treated with an end-to-end repair of an excised pseudoaneurysm with a vein graft taken from the radial artery vena comitantes through the same incision.

## Case presentation

**Figure 1:**
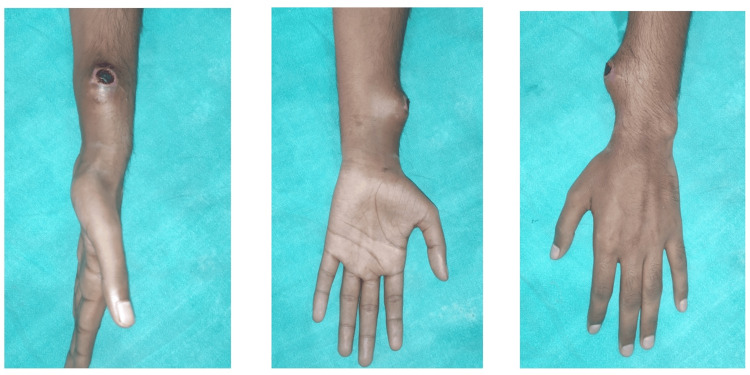
Preoperative picture of the radial artery pseudoaneurysm over the left wrist

A healthy 20-year-old male presented with progressively enlarging swelling near the left wrist over the lateral aspect for one month following a glass-cut injury. It was associated with a tingling sensation and difficulty in wrist or thumb movement due to pain. There was no history of fever or any other swelling in the body. On examination, a 3x3 cm oval swelling over the distal aspect of the left forearm on the radial side was seen 2 cm proximal to the radial styloid process with a scab over the apex of the swelling (Figure 1). No visible pulsation was present over the swelling. There was tenderness and increased temperature over the swelling. No palpable thrill was found, but radial and ulnar artery pulsations were present. Distal vascularity and sensation were present, and Allen’s test was negative. No bruit was heard. Ultrasonography findings were suggestive of an aneurysm of the distal left radial artery with a size 3.8x4 cm showing a "yin-yang" sign.

**Figure 2:**
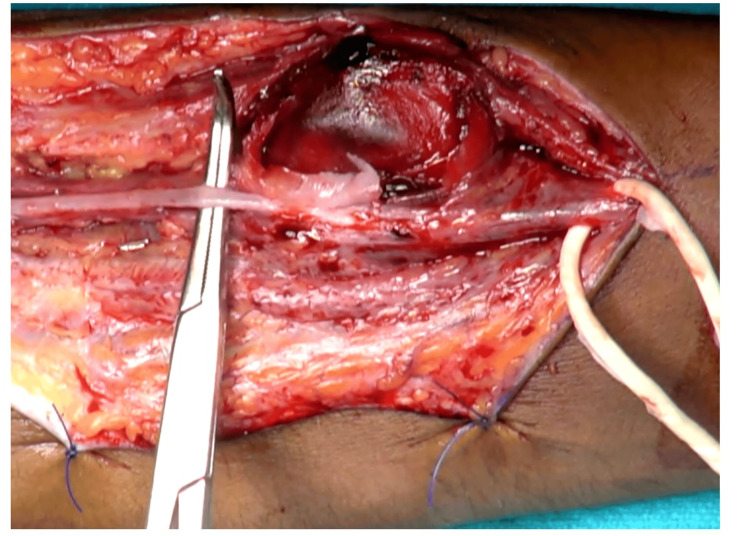
Intraoperative picture of the radial artery pseudoaneurysm

**Figure 3:**
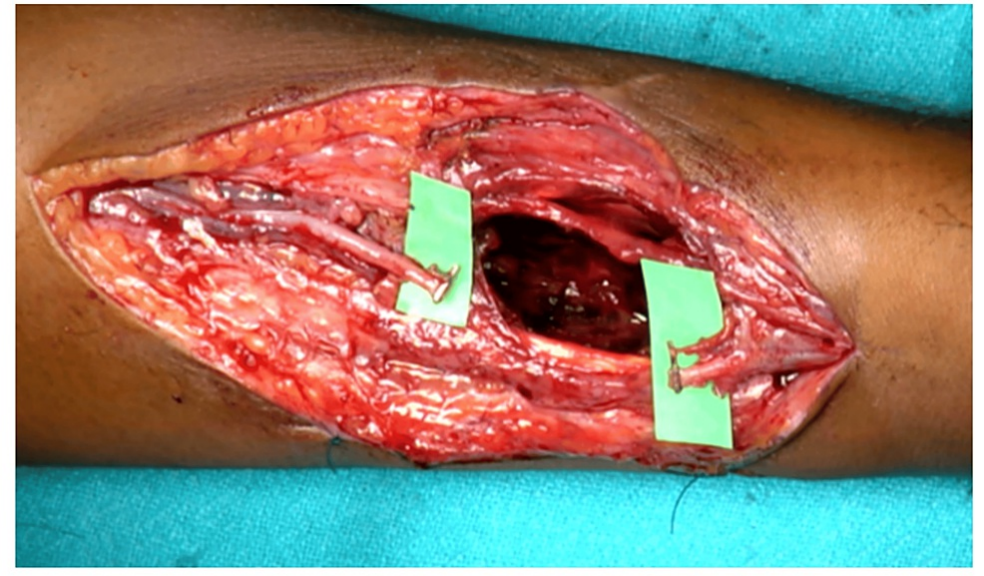
Cavity remaining after excising the pseudoaneurysm

**Figure 4:**
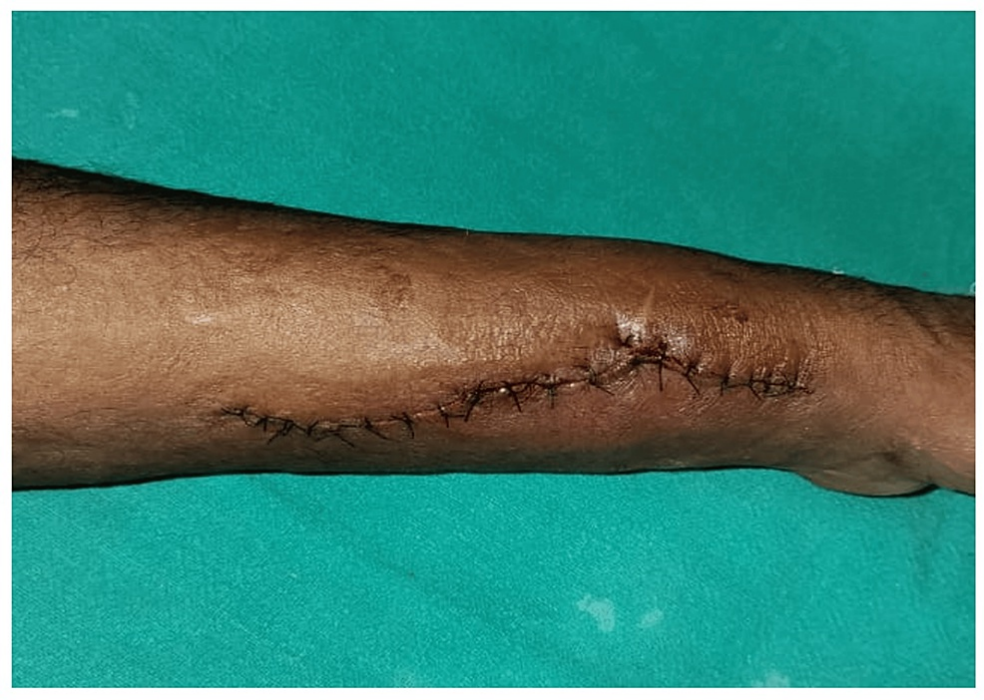
Postoperative picture after removal of the radial artery pseudoaneurysm

Through an incision just medial to the swelling, a 4x4x4 cm^3^ pseudoaneurysm of the left radial artery identified between the flexor carpi radialis and brachioradialis. The swelling was skeletonized (Figure 2). After securing proximal and distal vascular control, the mass was excised, and a huge cavity in the muscle plane and a 4 cm gap between two ends of the radial artery could be seen (Figure 3). The radial artery was repaired end to end with one of its venae comitantes grafts in a reverse pattern under magnification through a microscope and the space obliterated with a fibrillar, which being an absorbable hemostat not only helped in achieving hemostasis but also obliterated the dead space reducing chances of seroma formation. After closure, tight compression was applied. There was minimal serosanguinous discharge on the second day, which resolved with further dressings (Figure 4). Alternate-day dressing was done without administration of any anticoagulant, and the patient was discharged on the seventh postoperative day. Sutures were removed on the 14th postoperative day. Splintage was continued for three weeks. There was good blood circulation, and Allen’s test was negative after one month. No visible swelling was appreciated. Signed consent was obtained for publication from the patient.

## Discussion

Post-traumatic radial artery pseudoaneurysm is increasing in incidence. The reported incidence of pseudoaneurysm formation after transradial cardiac catheterization is up to 1% [2], but as it is seldom reported, its real incidence is unknown. It is formed following injury to all three layers of an arterial wall and accumulation of hematoma outside the arterial wall. Later, due to fibrin lysis, there is a formation of continuity with the arterial lumen. It usually occurs in the distal part of the vessel near the wrist, where it traverses superficially in between the tendons of the flexor carpi radialis (FCR) medially and brachioradialis laterally [3].

The clinical presentation might range from a locally increasing swelling to a painful mass with neurologic symptoms, pulsatile mass, and mass with necrosed skin or with a ruptured pseudoaneurysm [4]. It may appear within hours to months.

Pseudoaneurysms should be included as a differential diagnosis of the wrist mass, and puncturing of such a mass blindly should be avoided. Other differentials include hemangioma, ganglion cyst, seroma, hematoma, neurilemoma, arteriovenous fistula, epidermoid inclusion cyst, sarcoma, and Kawasaki disease [5].

Angiography is the gold standard for diagnosing pseudoaneurysms. As B-mode and color Doppler ultrasound are easily accessible, inexpensive, non-invasive, and radiation-free, they are the initial choice for the diagnosis of the condition. The classical sign of a pseudoaneurysm is due to its turbulent flow, called the “yin-yang” sign [6].

This condition should be attended to promptly due to local and systemic complications, such as embolism, skin erosion, thrombosis, bleeding, infection, pain, and paraesthesia, as a result of nerve compression. Although there is no clear-cut standard protocol, conservative, interventional, and surgical management is available. Encircling the limb with bandages, compression with an ultrasonography (USG) probe, ultrasound-guided thrombin injection, and surgical (ligation or reconstruction) treatment are done [7].

**Figure 5:**
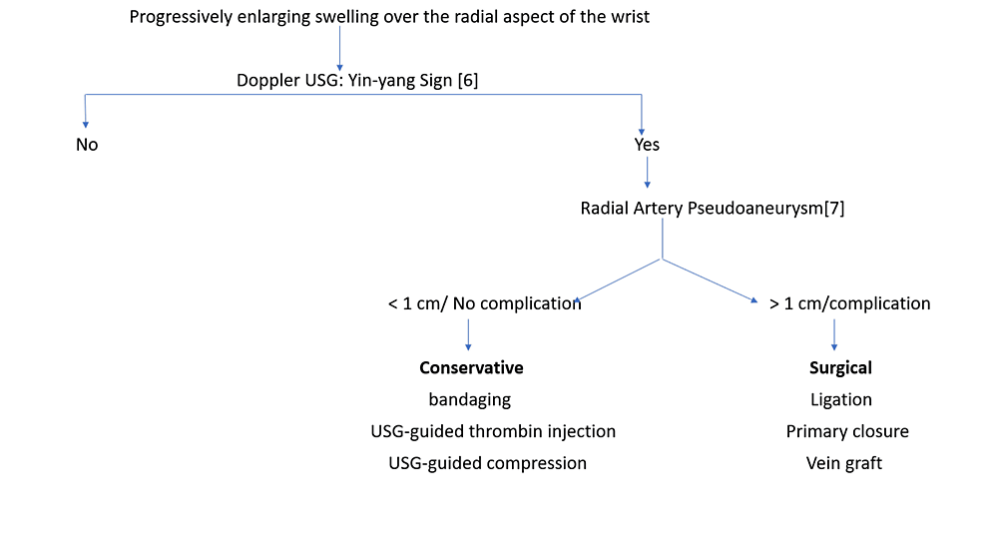
Algorithm for radial pseudoaneurysm management

Small-size pseudoaneurysms can be treated nonsurgically through bandaging, USG-guided thrombin injection, or USG-guided compression. Greater size (for adults >1 cm [7]), failure of conservative treatment, or complications require surgical planning (Figure 5).

There are various conservative methods; one of them is thrombin injection. Directly injecting thrombin into the pseudoaneurysm leads to thrombosis and eventually collapse of the pseudoaneurysm. There are several side effects, such as pain, allergy, necrosis, peripheral thrombosis, and arterial embolization, which can lead to loss of limbs. Ultrasound probe compression is another way to treat pseudoaneurysms. The primary objective is to form thrombosis inside the pseudoaneurysm by directly pressing an ultrasound probe on the swelling for 10-minute intervals until the swelling is totally occluded and compressed. Although this procedure has low complication rates, the implementation time of this procedure is long [8]. Both the techniques of directly injecting thrombin into the lesion and ultrasound probe compression may be done together.

Various surgical strategies may be employed that need to be individualized based on the size of the artery and collaterals. The artery may be ligated or repaired primarily or with autologous vein grafts, such as the great saphenous vein.

The radial artery may be ligated, but there are chances of tissue ischemia in the absence of good collateral circulation. Moreover, the hand would survive on one arterial supply if some trauma is to happen to that artery in the future, and the hand may be critical in survival.

In this study, with the swelling being a large one and greater than 1 cm along with complication of ulceration, surgical modality was chosen as the treatment option, and end-to-end repair of the excised pseudoaneurysm with an interposition vein graft taken from the radial artery vena comitantes was done through the same incision, which was previously reported by Simmons et al. [9]. This was possible as the size of the venae comitantes was comparable to the artery. As the vein was taken through the same incision, it decreased the number of scars and related complications. Two anastomoses were done between the radial artery vena comitantes and radial artery as the vena comitantes was interposed in the excised pseudoaneurysm. This was done under magnification through an operating microscope. There was a huge cavity left behind in the muscle compartment, which was filled with fibrillar decreasing the propensity of seroma formation.

## Conclusions

Post-traumatic radial artery pseudoaneurysm is a rare entity that can be treated non-surgically or surgically based on various factors. Based on the distance between two ends, it may be repaired primarily, or an interposition graft may be kept. If there is not much size discrepancy, the radial artery vena comitantes is a good option as an interposition graft with excellent results.
